# Mapping and Tracking Forest Burnt Areas in the Indio Maiz Biological Reserve Using Sentinel-3 SLSTR and VIIRS-DNB Imagery

**DOI:** 10.3390/s19245423

**Published:** 2019-12-09

**Authors:** Shou-Hao Chiang, Noel Ivan Ulloa

**Affiliations:** 1 Center for Space and Remote Sensing Research, National Central University, No. 300, Zhongda Rd., Zhongli District, Taoyuan City 32001, Taiwan; 2 Department of Civil Engineering, National Central University, No. 300, Zhongda Rd., Zhongli District, Taoyuan City 32001, Taiwan; ulloa_noel@hotmail.com

**Keywords:** wildfire detection, Sentinel-3 sea and land surface temperature radiometer (SLSTR), visible infrared imaging radiometer suite (VIIRS) day night band, image segmentation, Indio Maiz Biological Reserve

## Abstract

Wildfires are considered one of the most major hazards and environmental issues worldwide. Recently, Earth observation satellite (EOS) sensors have proven to be effective for wildfire detection, although the quality and usefulness of the data are often hindered by cloud presence. One practical workaround is to combine datasets from multiple sensors. This research presents a methodology that utilizes data of the recently-launched Sentinel-3 sea and land surface temperature radiometer (S3-SLSTR) to reflect its applicability for detecting wildfires. In addition, visible infrared imaging radiometer suite day night band (VIIRS-DNB) imagery was introduced to assure day-night tracking capabilities. The wildfire event in the Indio Maiz Biological Reserve, Nicaragua, during 3–13 April 2018, was the study case. Six S3-SLSTR images were processed to compute spectral indices, such as the normalized difference vegetation index (NDVI), the normalized difference water index (NDWI), and the normalized burn ratio (NBR), to perform image segmentation for estimating the burnt area. The results indicate that 5870.7 ha of forest was affected during the wildfire, close to the 5945 ha reported by local authorities. In this study, the fire expansion was delineated and tracked in the Indio Maiz Biological Reserve using a modified fast marching method on nighttime-sensed temporal VIIRS-DNB. This study shows the importance of S3-SLSRT for wildfire monitoring and how it can be complemented with VIIRS-DNB to track burning biomass at daytime and nighttime.

## 1. Introduction

Wildfires are considered a major hazard and environmental problem around the world [1,2,3]. The significant ecological damage of a forest fire is caused by a large amount of greenhouse gases emitted into the atmosphere by the burning biomass [4], the accelerated soil erosion rate, and changes in the physical, chemical, and biological properties of soil [5,6]. In Latin America, forest fires are considered one of the main causes of deforestation and forest degradation [7].

On 3 April 2018, a fire broke out inside the Indio Maiz Biological Reserve, which is part of the Rio San Juan Biosphere Reserve, in the southeast of Nicaragua [8,9]. After days of continuous and arduous work from the local authorities, the fire was extinguished in the afternoon of 13 April (local time) [10,11]. Reports from the national government indicate that a total of 5945 ha was burnt in this event, which is now considered as one of the worst ecological disasters ever seen in the country [12]. Due to the remoteness of the area and lack of accessibility, it is difficult to make estimations of the magnitude and extension of the fire based on in situ data, especially during its initial stages. Therefore, remotely-sensed imagery is highly recommended to visualize and assess the impact of wildfire events. This emphasizes the role of Earth observation satellites (EOSs), which are capable of providing emergency response teams with crucial information, such as burnt areas, ignition points, and movement of the fire rim.

The moderate resolution imaging spectroradiometer (MODIS), the land remote-sensing satellite program (Landsat), and the visible infrared imaging radiometer suite (VIIRS) are some of the most commonly used EOSs for forest fire detection, monitoring, and assessment on a regional and global scale [13,14,15,16]. The variation of spatial and temporal resolutions of EOS sensors is a critical issue for fire detection. Most importantly, the revisit frequency and timing of satellites usually affect the effectiveness of emergency monitoring. A practical workaround is to combine multiple sensors of different spatial and temporal resolutions to produce rapid and reliable estimations regarding the location and extent of wildfires in tropical zones. Reducing the time required to identify wildfire can lead to increased situational awareness, as well as faster decision making by fire control teams [17]; thus reducing the potential negative impact that an outbreak fire could have on areas with high biodiversity and fragile ecosystems (e.g., the Indio Maiz Biological Reserve).

The sea and land surface temperature radiometer (SLSTR) sensor on board of the Sentinel-3A and 3B satellites is the newest addition to the lineup of orbiting spaceborne optical sensors. These satellites were launched by the European Space Agency (ESA) in February 2016 and 8 April 2018, respectively [18]. These twin satellites provide images with 500 m and 1 km spatial resolution over a swath of 1420 km in the visible, infrared, and thermal wavelength, with a temporal resolution of 1 day at the equator [19,20]. The Sentinel-3 SLSTR (S3-SLSTR) sensor possesses dedicated bands for fire detection, and the high temporal and spectral resolution gives it high potential for rapid detection and tracking of forest fires, as well as for the systematic monitoring of the recovery process of burnt areas [21]. In addition, day time monitoring of land surface may be complemented with data acquired during the night time by the visible infrared imaging radiometer day Nightn band (VIIRS-DNB), provided by the National Oceanic and Atmospheric Administration (NOAA). VIRRS-DNB has high sensitivity to low light sources [22,23]. It is capable of detecting biomass burning at night in the visible spectrum [21,24].

For this study, a single forest fire event that took place in the Indio Maiz Biological Reserve, Nicaragua, during 3–13 April 2018, was selected as the study case. The objective of this study is to demonstrate the potential of the S3-SLSTR sensor for active wildfire detection and burnt area estimations over protected forest area and to show the VIIRS-DNB capability of reconstructing the propagation of the fire rim.

To better capture the evolution of the wildfire event in the study area, this research presents a new methodology of day/night monitoring wildfire events by employing optical data from the S3-SLSTR and VIIRS-DNB sensor data record (SDR), which was acquired between 00:00–02:00 AM local time (UTC-6). Spectral indices, such as the normalized difference vegetation index (NDVI), the normalized difference water index (NDWI), and the normalized burn ratio (NBR), were derived to perform image segmentation for estimating burnt areas. Comparisons of burnt area estimated by the MODIS and Sentinel-2 were also made to corroborate the fire-detection capabilities of S3-SLSTR imagery and to have a better evaluation of rapid wildfire response and real-time wildfire detection for the Indio Maiz Biological Reserve wildfire event. In addition, VIIRS-DNB scenes were digitally enhanced to increase the visibility of dim light sources, while preserving detail in brighter areas. With integrating the VIIRS active fire product, a modified fast marching method (FMM) [25,26] was then applied on the nighttime-sensed data to delineate the extension of the active fire. We suggest that the time-series of VIIRS-DNB complements the results obtained from traditional optical sensors to provide daytime and nighttime wildfire tracking capabilities.

## 2. Study Area and Satellite Data

### 2.1. Study Area

**Figure 1:**
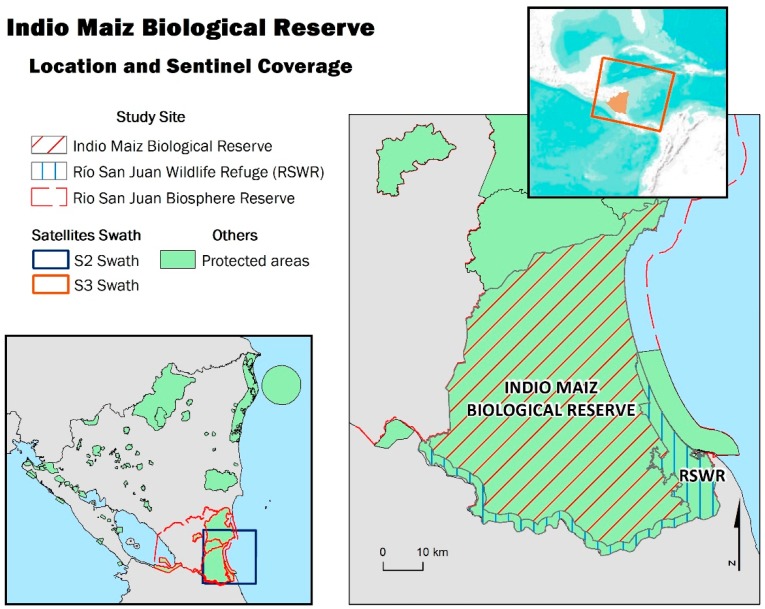
Coverage of Sentinel-3 (S3 Swath) in the upper right, coverage of Sentinel-2 (S2 swath) in the lower left, and location of the Indio Maiz Biological Reserve on the right.

The study area was the Indio Maiz Biological Reserve, located in the southeast of the Republic of Nicaragua, Central America (Figure 1). It has an extension of 263,980 ha [27] and it is one of the seven protected areas that conform to the Rio San Juan Biosphere Reserve (1,392,900 ha) [28]. The Indio Maiz Biological Reserve is considered one of the largest and most pristine protected areas in the Central American region, in part thanks to its remote location, difficult accessibility, and low population. This reserve is also part of the Mesoamerican Biological Corridor (MBC), an area of high biodiversity created in the late 1990s with the aim of preserving the biological connectivity and protecting threatened and endangered wildlife [29,30,31]. The forest habitat of the Indio Maiz Biological Reserve possesses abundant biodiversity and is home to endangered and vulnerable bird and mammal species, such as *Harpia harpyjia*, *Ara ambigua*, *Electron carinatum*, and *Tapirus bairdi bairdi* [29,32,33].

The well-conserved ecosystems of the study area are under pressure from development projects and illegal farming activities. Despite its status as a protected area, cattle ranching activities in the reserve have been on the rise: Up to 6.5% of the national farming activity takes place in this area [34,35]. The occurrence of natural or man-induced wildfires are uncommon in the core area of the Indio Maiz Biological Reserve; however, the increasing number of colonists who have settled in the reserve poses a significant threat to the conservation of this area. To slash-and-burn is a common practice among these people, in order to clear the forest land and convert it into more profitable land uses (e.g., agricultural, cattle ranching), thus increasing the risk of human-induced fire outbreak.

### 2.2. Applied Satellite Imagery

#### 2.2.1. MODIS Terra

For this study, the MODIS Terra Surface Reflectance product (MOD09GQ V006) was downloaded from the online Data Pool (https://ladsweb.modaps.eosdis.nasa.gov/), courtesy of the National Aeronautics and Space Administration (NASA) Land Processes Distributed Active Archive Center (LP DAAC). Images were acquired for the period between 3–18 April 2018. MOD09GQ products are available over one or two days with a spatial resolution of 250 m across the red (620–670 nm) and near-infrared (841–876 nm) spectral bands [36]. These bands are corrected for the atmospheric effect of gases, aerosols, and Rayleigh scattering. The MODIS data was projected from Sinusoidal projection to Universal Transverse Mercator (UTM) projection WGS84 coordinates.

The normalized difference vegetation index (NDVI) was derived from the red and near-infrared band. Afterward, a composite was created using the red, infrared, and NDVI bands for each of the scenes acquired.

#### 2.2.2. Sentinel-2 and Sentinel-3 Optical Imagery

Sentinel-2A and 2B were part of the European Copernicus program. Each satellite was equipped with a multi spectral instrument (MSI) that acquires data from the visible and near-visible spectrum over a swath of 2 km, with a spatial resolution that ranges from 10 to 60 m, depending on the spectral band [37,38]. An image can be acquired by either satellite every 5 days [39]. A total of four images were obtained from the European Space Agency (ESA) Copernicus Open Access Hub (https://scihub.copernicus.eu/dhus/) for the period 6–26 April 2018. The scenes were acquired as top-of-atmosphere level 1C images. The red band was resampled using the bilinear interpolation resample method to reach the same spatial resolution as the short-wave infrared (SWIR) bands. The images were then re-projected to projected coordinates, UTM Zone 16 N, WGS84.

The Sentinel-3A satellite was launched on 16 February 2016 [40] as the newest element of the Copernicus program. Two of the payloads it carried were the ocean and land colour instrument (OLCI) and the sea and land surface temperature radiometer (SLSTR) [41]. The latter sensor includes visible (VIS) to thermal infrared (TIR) channels that provide radiance measurements suitable for active fire detection at a resolution of 500 m (VIS, SWIR) and 1 km (TIR) [42,43,44].

Sentinel-3A SLSTR data was downloaded as the level-1 radiance and brightness temperature (SL_1_RBT) from the Copernicus Online Data Access (CODE; https://coda.eumetsat.int/#/home), corresponding to the period 4–23 April 2018. The data was preprocessed using the SNAP Sentinel-3 toolbox (https://coda.eumetsat.int/#/home): The images were projected to UTM WGS 84 and the radiance was converted to top-of-atmosphere reflectance. A composite of the bands with 500 m spatial resolution was created and applied in this study.

Table 1 summarizes the availability of scenes from a MODIS, a Sentinel MSI, and a SLSTR during the duration of the wildfire at the Indio Maiz Biological Reserve. The cloud coverage condition over the study site is also an important issue in the analysis. When clouds covered the affected zone and led to an unsuccessful burnt area estimation (see next section), the image was labeled as “available but contaminated”, as shown in Table 1. Moreover, we also included the first cloud-free post-event imagery in the burnt area estimation for each sensor: 18 April, 23 April, and 26 April for the MODIS-Terra, S2-MSI, and S3-SLSTR, respectively.

#### 2.2.3. VIIRS Day Night Band

VIIRS incorporates the radiometric accuracy of the advanced very high resolution radiometer (AVHRR), along with improved spectral and spatial resolution. VIIRS includes 22 spectral bands. The 17th moderate resolution day night band is a broad (0.5–0.9 μm) band, which can be used to collect nighttime visible/near-IR image data at a resolution of 0.75 km across the entire scan width. VIIRS-DNB sensor data record (SDR) files from 3–14 April 2018 were used to create a time-series of the fire event and analyze the spread of the fire ring. This dataset was provided by the National Oceanic and Atmospheric Administration (NOAA), through its comprehensive large array-data stewardship system (CLASS) website (https://www.class.ngdc.noaa.gov/saa/products/catSearch). Multiple DNB bands are available for each day at different sensing times. The DNBs selected for this study were acquired between 06:00–08:00 Universal Time Coordinated (UTC) time, corresponding to 00:00–02:00 in local time.

## 3. Wildfire Monitoring

### 3.1. Burnt Area Estimation

**Figure 2:**
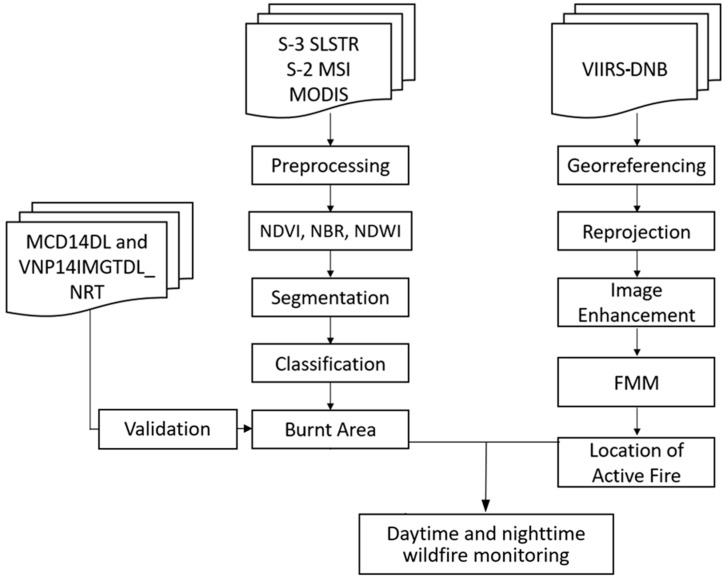
Workflow for wildfire detection and monitoring in the Indio Maiz Biological Reserve. Sentinel-3 sea and land surface temperature radiometer (S3-SLSTR); multi spectral instrument (MSI); moderate resolution imaging spectroradiometer (MODIS); visible infrared imaging radiometer suite day night band (VIIRS-DNB); normalized difference vegetation index (NDVI); normalized burn ratio (NBR); normalized difference water index (NDWI); fast marching method (FMM).

As depicted in Figure 2, after preprocessing the acquired images, the following step was to derive spectral indices commonly used to identify burnt areas in satellite images. Since Sentinel-2 MSI and Sentinel-3 SLSTR imagery possess visible and infrared bands with similar bandwidth, we calculated the normalized difference vegetation index (NDVI) [45,46], normalized difference water index (NDWI) [47,48], and normalized burn ratio (NBR) [49,50] for each scene of Sentinel-2 and Sentinel-3 (Table 2).

The next step was to create a composite with the red, near infrared (NIR), shortwave infrared (SWIR), and SWIR 2 bands, along with the spectral indices, and perform the segmentation of each scene using the eCognition Developer [51]. In the case of the MODIS, the composite was created using the red and NIR bands, plus NDVI. The segmentation of the images allows us to aggregate pixels with similar spectral characteristics into objects that are easier to analyze and interpret than individual pixels [52,53]. The segmentation parameters had to be adjusted for each satellite product due to their different spatial resolutions, as shown in Table 3.

After segmentation, the objects near the reported fire site were visually interpreted and merged into larger objects and labeled as a burnt area. The fire scar detected in all the scenes for each dataset were then merged to obtain a total accumulated burnt area. The burnt areas which could not be successfully estimated by images with cloud contamination (see Table 2) were excluded, and cloud-free images after the fire event were also analyzed to assure the completeness of the burnt area estimation.

Finally, the different burnt areas calculated from the MODIS, Sentinel-2, and Sentinel-3 were compared to one another and to the official burnt area reported by local authorities to assess the feasibility of Sentinel-3 SLSTR data for the burnt area estimation.

### 3.2. Validation with Active Fire Data

The mapped burnt scars were validated using the MODIS (MCD14DL) and VIIRS (VNP14IMGTDL_NRT) active fire products acquired from the Fire Information for Resource Management System (FIRMS) website (https://earthdata.nasa.gov/earth-observation-data/near-real-time/firms/active-fire-data), for the period 3–11 April 2018.

MODIS MCD14DL products are generated by applying thresholds in the middle and thermal infrared bands [54] yielding 1 km detections. Similarly, the VIIRS fire detection products are created using aforementioned bands, but iprovide data at a finer resolution (350 m), improving the detection of active fire over smaller areas [55,56].

### 3.3. VIIRS-DNB Fire Spot Delineation

To track the nighttime wildfire dynamics, VIIRS-DNB scenes were digitally enhanced to increase the visibility of dim light sources. The VIIRS active fire product and a modified fast marching method (FMM) [25,26] were then integrated and applied for delineating the extension of the active fire. Because the VIIRS-DNB possesses a dynamic range of approximately 7 orders of magnitude [57], a square root stretch was applied to the data in order to normalize the values so that dim light sources could become visible without losing detail in brighter areas. The implementation of the image enhancement went according to the following procedure:

*E*_i,j_ = min{[(*DNB*_i,j_ + *a*) × *b*]^0.5^, 2^16^ − 1} (1)

where *E*_i,j_ represents each pixel in the enhanced image; *DNB*_i,j_ is the pixel from the geometrically corrected DNB; *a* is an offset to mitigate the presence of small sporadic negative values in the original dataset; and *b* is a coefficient to fit the data into a 16-bit depth image. Any pixel whose value was higher than 65,535 (2^16^ − 1) was set to 65,535, since this is the maximum value that can be stored in a 16-bit image.

Once the images were enhanced, we proceeded to detect the boundary of the area affected by the wildfire. The fast marching method (FMM) is a simple and computationally efficient numerical method used for solving boundary problems, such as the motion of a propagating front. Previously, this method has been applied to numerous fields, such as road extraction from satellite imagery [58], path planning [59,60], and medical image segmentation [61]. In this work, we treated the active fire visible in the DNB as a region with a defined boundary, and, by setting a seed point inside the area, FMM could segment the image and label each pixel as inside or outside the object based on a set threshold [62]. In this case, the VIIRS active fire product is an ideal seed point to initiate the segmentation, given the fact that this dataset represents the location of hotspots with high thermal output. Depending on the extent of the wildfire event, multiple points may be available for the same date. In such scenarios, we selected the points with the highest fire radiative power (FRP).

## 4. Results

### 4.1. Burnt Area Estimation and Forest Fire Tracking

**Figure 3:**
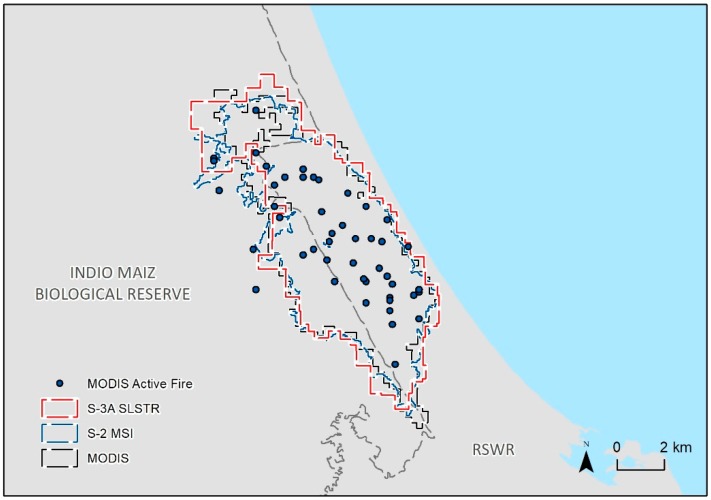
Distribution of the MODIS active fire and burnt area derived from MODIS Terra, Sentinel-2 MSI, and Sentinel-3A SLSTR.

Figure 3 displays the total accumulated burnt area derived from the MODIS and the optical Sentinel satellites, as well as the spatial coherence with the MODIS active fire products. The total accumulated burnt area estimations are shown in Table 4. In general, the MODIS-derived burnt area was the smallest, whereas S3-SLSTR yielded the highest estimation. The differences in the total burnt area values among the three datasets are mainly due to the cloud cover issue and different spatial resolution.

Through aerial image analysis, the national authorities of Nicaragua reported a total burnt area of 5945 ha, affecting the Indio Maiz Biological Reserve and the Rio San Juan Wildlife Refuge [63]. The reported area is greater than the estimated value from MODIS-Terra, S2-MSI, and S3-SLSTR. The mapping accuracy of the burnt area distribution was not assessed because the ground truth data is not available. However, the results indicate that the burnt area mapped by S3-SLSTR is generally consistent with MODIS-Terra and S2-MSI (Figure 3); therefore, it is possible to use this new dataset to produce close estimations of forest areas affected by the fire with a high temporal resolution, even in cases where the burning area is confined to a small zone.

The maps derived from S2-MSI and S3-SLSTR show an area coherence of 78%, and the total combined burnt area detected by both satellites was 6198.4 ha. The coherence obtained within this study was higher than that reported in the previous work by Verheggen et al [64]. The authors derived burnt area maps for the Congo Basin forest for the period of November 2015 to May 2016, using Sentinel-1 and Sentinel-2 imagery. They reported a total of 36,247 ha affected by fire, with a spatial coherence of 68% for the two datasets. Compared to the combination of optical and synthetic aperture radar (SAR) data, Sentinel-2 and Sentinel-3 complementary application provides another benefit: Faster and considerably less complex processing that needs to be carried out to analyze the data, which is of critical importance for fast response teams during emergency events.

**Figure 4:**
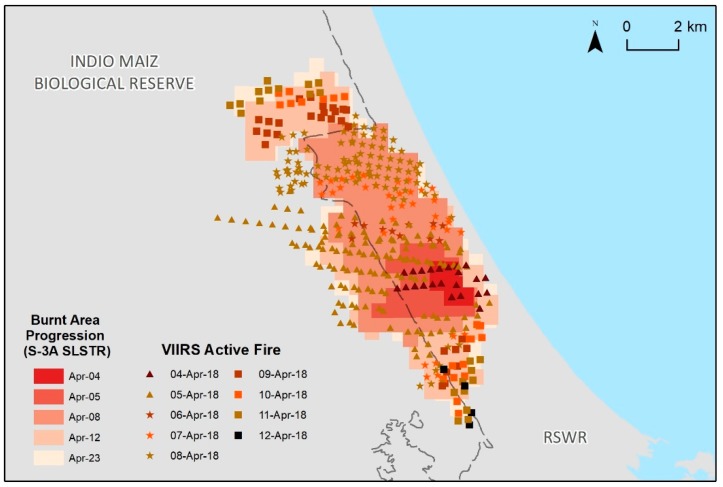
Comparison of the Sentinel-3A SLSTR-derived burnt area and the VIIRS active fire product.

The VIIRS active fire product corresponding to 3–14 April is also shown in Figure 4. The high spatial correlation between this product and the S3-SLSRT-derived burnt area confirms the usefulness of S3-SLSTR for wildfire monitoring. Color coding is used in Figure 4 to highlight the origin and expansion of the burn-scar from 4 to 23 April, as detected on S3-SLSRT imagery.

For the application of multi-sensors in this study, Sentinel-2 and MODIS-Terra had the same overpass time (10:30 AM), whereas Sentinel-3 flew over the center scene at 10:00 AM local time. Although the difference in acquisition time between MODIS-Terra and Sentinel-3 is limited, considering the cloud conditions over a site could possibly vary significantly in short periods of time, we suggest that the use of S3-SLSRT data in conjunction with other datasets is still able to increase the possibility of acquiring useful data for active fire monitoring.

### 4.2. VIIRS-DNB Active Fire Time Series

**Figure 5:**
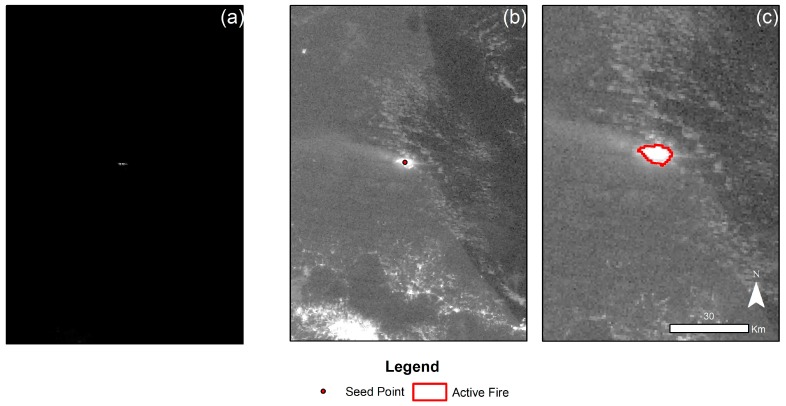
Enhancement of VIIRS-DNB and active fire spot delineation. VIIRS-DNB from 5 April. The seed point corresponds to VNP14IMGTDL_NRT for the same date: (**a**) Image before enhamcement, (**b**) Image after enhacement, (**c**) Delineated active spot using FMM.

The broad dynamic range of the original VIIRS-DNB implicates that the difference between sources of dim light and bright areas can be several orders of magnitude. As a result, small-scale wildfires may not be clearly appreciated in the imagery (see Figure 5a), unless it is enhanced.

Our image enhancement results clearly show improvement of the visibility of the active fire compared to the original dataset. The square root stretch was able to increase the intensity of dim areas while improving the visibility of background features (Figure 5b). We also show the spatial coherence between the bright spot visible in the DNB and the VIIRS active fire product for the same date, indicating that such areas correspond to an ongoing wildfire. Furthermore, by using the FMM segmentation, it was possible to estimate the extent of the active fire in the DNB imagery, as shown in Figure 5c.

**Figure 6:**
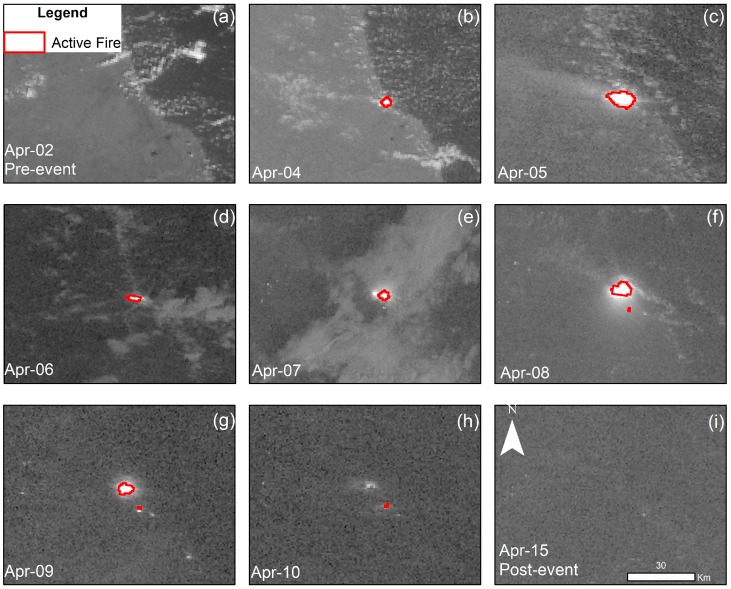
Enhanced VIIRS-DNB time series shows the progress of the fire rim inside the Indio Maiz Biological Reserve from 2 to 18 April 2018: (**a**) 2 April (**b**) 4 April, (**c**) 5 April, (**d**) 6 April, (**e**) 7 April, (**f**) 8 April, (**g)** 9 April, (**h**) 10 April, and (**i**) 15 April.

We replicated this process in all available DNB scenes available where the wildfire was visible. The detection of the active fire and its expansion is shown as a chronoseries in Figure 6, from its onset until its containment.

In Figure 6, the fire is first visible on the image acquired on 4 April (Figure 6b), at 00:37 local time, more than 7 h after the fire started. At first, the fire spread in the westward direction (Figure 6c), and then it continued its advance to the north (Figure 6d). In subsequent images (Figure 6e,f), it can be perceived that the two fire rims formed when the main fire rim had a north trajectory and a smaller fire rim spread in the south. Furthermore, in Figure 6g,h, it is appreciable how the fire reduced its intensity because of the intervention of the emergency response teams. According to the national authorities, a rain event on 12 April that took place in the area helped suffocate the remaining active fire. In Figure 6i, no active fire or smoldering zones were detected by the VIIRS-DNB.

This sequence of images confirms the applicability of the VIIRS-DNB in an emergency situation, such as monitoring, managing, and analyzing on-going wildfire events. Besides detecting active burning biomass, it is also able to observe the smoke plume, usually undetected by thermal sensors, caused by the wildfire in some of the scenes (Figure 6c,f). This information is extremely critical to forecast possible wind shifts or circulations triggered by the fire [24], thus helping to increase the security of the emergency response teams.

## 5. Discussion

### 5.1. Applicability of Sentinel-3-SLSTR for Wildfire Monitoring

**Figure 7:**
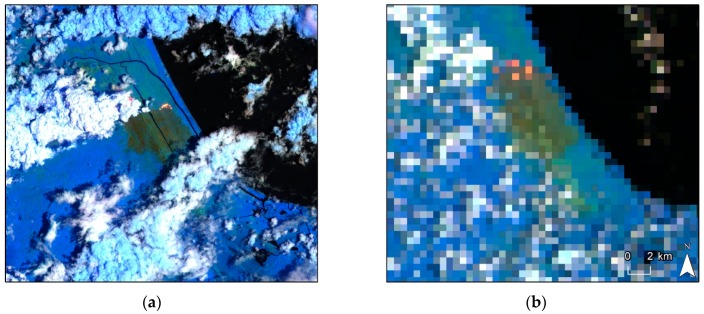
Atmospheric penetration composite for (**a**) Sentinel-2 MSI and (**b**) Sentinel-3 SLSTR.

By combining the Sentinel-3 NIR, SWIR, and SWIR-2 bands, it is possible to create a false-color composite capable of penetrating atmospheric particles, smoke, and haze. In addition, the use of the shortwave infrared bands makes it possible to detect areas with high heat output, such as active fires. Figure 7 shows the atmospheric penetration composite for S2-MSI and S3-SLSRT while the wildfire was still active.

A side-by-side comparison (Figure 7) confirms that due to the similar radiometric resolution, imagery from the S3-SLSTR is capable of identifying zones with active fire. This information is crucial for emergency response teams, even more so if the fire is detected in its early stages, to estimate and evaluate the future spread of a wildfire, in order to design fast-response and evacuation plans of nearby communities if necessary.

Moreover, the Sentinel-3 SLSTR also has the capability of detecting the burn scars by combining bands 6,3,2 (SWIR, NIR, and Red), as seen in Figure 6. In these images, the recent burn areas appear in a brown tone. This estimation is possible because of the lasting spectral signal emitted by the charcoal residue. The information that can be obtained from S3-SLSTR could be used to evaluate the environmental and economic damage produced by the wildfire and estimate the recovery of burnt areas.

Although the coarse resolution of S3-SLSTR can be considered a limitation when analyzing fire events with small extension, its high temporal resolution makes it suitable to track and keep record of areas affected by wildfires. In order to make a substantial improvement in fire-fighting decision making, a high frequency of observation is needed [17].

S3-SLSTR data represents another tool that emergency response teams can utilize, as well as another source of information for post-event delineation and monitoring of burn areas. This after-event analysis, combined with meteorological data, can provide some insight into the wildfire spread dynamics and improve wildfire prediction and prevention efforts. Cloud cover is still a considerable issue for delivering accurate data on forest fire events, a fact that highlights the benefits of having an additional spaceborne sensor capable of monitoring such hazards.

**Figure 8:**
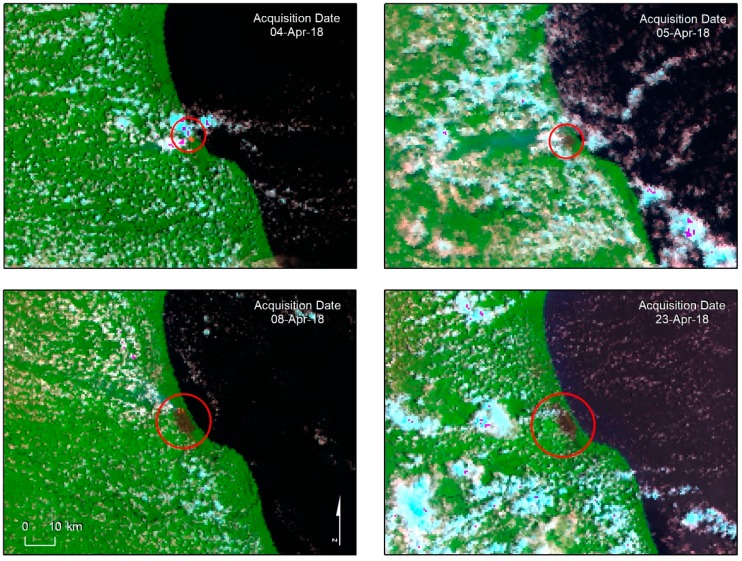
Tracking of burn scar expansion using the Sentinel-3 SLSTR from 4 to 23 April 2018.

In Figure 8, the evolution of the burn scar obtained from the SLSTR data is similar to what was detected in the VIIRS DNB analysis (Figure 6); after the fire started it propagated toward the west. The presence of the Atlantic Ocean east of the fire ignition point served as a natural barrier, limiting the direction to which the fire could spread. By 8 April, most of the burnt area was located in the northwest direction from where it had originated; however, the scenes acquired on 12 April and 23 April reveal that zones south of the origin had also been affected by this wildfire.

### 5.2. Forest Types Affected by the Wildfire Event

**Figure 9:**
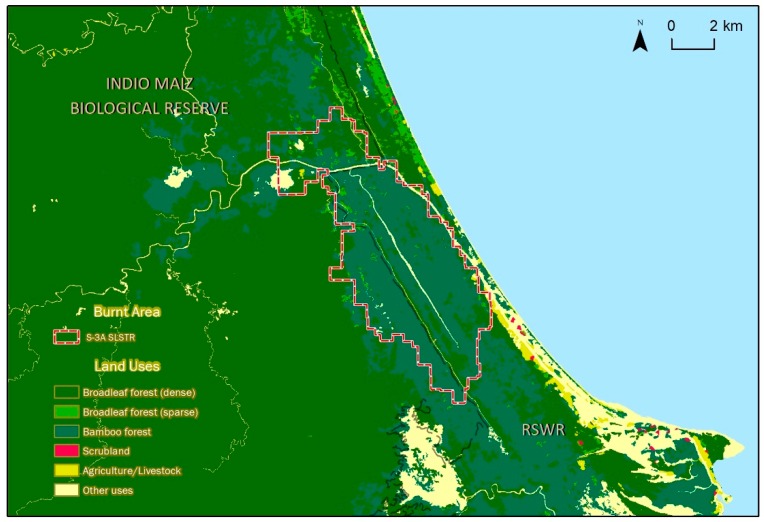
Forest types affected by the fire event in the Indio Maiz Biosphere Reserve.

The superposition of the remotely sensed burnt area over the forest map of 2015 reveals the main forest types affected by this event. Figure 9 shows that 73% of the burnt area belongs to a palm tree forest, followed by 20.4% of a broadleaf forest. Field investigation by the national authorities confirmed that in the aforementioned area, there is an abundance of one specific type of palm tree: yolillal (*Raphia taedigera*). Yolillal palm trees are native to the Americas and they are commonly present in the coastal ecosystem of the Nicaraguan Caribbean [32,65]. They are a food source for different animal species, and they also help regulate the water level in low elevation zones. More importantly, *Raphia taedigera* has a high content of oil, meaning that fire is easily and rapidly propagated in this type of forest. Figure 9 shows that the propagation of the blaze was almost constrained to areas covered by the palm tree forest. In this event, the fire relief efforts carried out by the authorities prevented the fire from continuing its path in the south direction, where a larger area of palm trees would have been affected.

Thanks to their deep roots, *Raphia taedigera* can regenerate rapidly as long as there is no intervention in the area. The use of S3-SLSTR data to monitor the recovery of the ecosystem in this zone could be also expected. Moreover, given the similarity in terms of the spatial, temporal, and spectral resolution between the MODIS and Sentinel-3 SLSTR, the latter may have other potential uses in the forestry field beyond wildfire monitoring, such as deforestation assessment, plague propagation in forested areas, and biomass estimation among others [66,67,68].

It is important to mention that the wildfire event focused in this study created a relatively isolated and small burn scar in comparison to other major wildfires that frequently take place in other parts of the world (i.e., California). The scale and diversity of affected vegetation and landscape are also relatively limited in this study case. Therefore, the proposed detection and monitoring techniques should be examined when testing other wildfire events with different scales, magnitude, and patterns.

## 6. Conclusions

This study corroborates the applicability of Sentinel-3 SLSTR data to detect and monitor the occurrence of wildfires in a tropical forest in the Indio Maiz Biological Reserve, Nicaragua, and to generate burnt area estimations with a high temporal resolution. The burnt area estimation was conducted by image segmentation using the normalized difference vegetation index (NDVI), the normalized difference water index (NDWI), and the normalized burn ratio (NBR). The analysis of SLSTR data reveals that 5870.7 ha of forest was affected during the wildfire event that took place in the study area in April 2018. This estimation is close to the 5945 ha reported by the local authorities. However, the uncertainty of the burnt area estimation can be attributed to image availability, the effect of cloud contamination in each scene, and limitation due to image resolution.

This study shows the Sentinel-3 SLSTR capability of generating fire-monitoring optimized images within 2–3 days, and it is complementarity with VIIRS-DNB data for daytime and nighttime active fire monitoring. The nighttime fire expansion was delineated and tracked using a modified fast marching method. Because the studied burnt area is relatively isolated and small, the scale and diversity of the affected vegetation and landscape are also limited. Therefore, we suggest the proposed detection and monitoring techniques should be further examined when testing other wildfire events with different scales, magnitude, and patterns.

## Figures and Tables

**Table 1:** Data availability and data quality for scenes available during the wildfire.

Sensor/Date	3 April	4 April	5 April	6 April	7 April	8 April	9 April	10 April	11 April	12 April	13 April
MODIS-Terra	abc	a	a	a	abc	a	abc	abc	abc	abc	abc
S2-MSI	na	na	na	a	na	na	na	na	a	na	na
S3-SLSTR	na	a	a	na	na	a	abc	na	abc	a	na

Available: a; Available but contaminated: abc; Not-available: na.

**Table 2:** Band ratios used for burnt area estimations.

Index	Equation	Corresponding Bands	
		S2-MSI	S3-SLSTR
NDVI	$\frac{\mathrm{NIR}-\mathrm{Red}}{\mathrm{NIR}+\mathrm{Red}}$	8a (NIR); 4 (Red)	S3 (NIR); S2 (Red)
NBR	$\frac{\mathrm{NIR}-\mathrm{SWIR}\;2}{\mathrm{NIR}+\mathrm{SWIR}\;2}$	8a (NIR); 12 (SWIR 2)	S3 (NIR); S6 (SWIR 2)
NDWI	$\frac{\mathrm{NIR}-\mathrm{SWIR}}{\mathrm{NIR}+\mathrm{SWIR}}$	8a (NIR); 11(SWIR)	S3 (NIR); S5 (SWIR)

**Table 3:** Segmentation parameters.

Data	Scale	Shape	Compactness
MODIS-Terrra	30	0.1	0.5
S2-MSI	5	0.1	0.5
S3-SLSTR	20	0.1	0.5

**Table 4:** Estimation of total accumulated burnt area.

Data	Accumulated Burnt Area (ha)	Spatial Resolution (m)
MODIS-Terrra	5033.7	250
S2-MSI	5187.3	20
S3-SLSTR	5870.7	500

## References

[1] Krstic N., Henderson S. (2015). Use of MODIS data to assess atmospheric aerosol before, during, and after community evacuations related to wildfire smoke. Remote Sens. Environ..

[2] Chen G., He Y.A., de Santis A., Li G.S., Cobb R., Meentemeyer R.K. (2017). Assessing the impact of emerging forest disease on wildfire using Landsat and KOMPSAT-2 data. Remote Sens. Environ..

[3] Fernandez-Garcia V., Santamarta M., Fernandez-Maso A., Quintano C. (2008). Burn severity metrics in fire-prone pine ecosystems along a climatic gradient using Landsat imagery. Remote Sens. Environ..

[4] Urbanski S., Salmon J., Nordgen B., Hao W. (2009). A MODIS direct broadcast algorithm for mapping wildfire burned area in the western United States. Remote Sens. Environ..

[5] Zavala L., de Celis R., Jordan A. (2014). How wildfires affect soil properties: A brief review. Geogr. Res. Lett..

[6] Conrado M., Chang K., Chen C., Chiang S., Santos J. (2016). Modelling the spatial variability of wildfire susceptibility in Honduras using remote sensing and geographical information systems. Geomat. Nat. Hazards Risk.

[7] Julio-Alvear G. Managing efforts to prevent forest fires in South America. Proceedings of the Second International Symposium on Fire Economics, Planning, and Policy: A Global View.

[8] Villavicencio F., Salazar M. (2018). Fuego Podría Extenderse al Límite de Reserva Indio Maíz. https://confidencial.com.ni/.

[9] Torrez C. (2018). Ambientalistas Sospechan de Mano Criminal en Incendio en la Reserva Indio Maíz. https://www.laprensa.com.ni/2018/04/05/nacionales/2399577-incendio-devora-reserva-indio-maiz/.

[10] Romero E., Torrez C. (2018). Fuego Arrasó con más de 5000 Hectáreas en la Reserva Indio Maíz. https://www.laprensa.com.ni/2018/04/13/nacionales/2403540-gobierno-asegura-que-se-termino-el-incendio-en-la-reserva-indio-maiz/.

[11] (2018). Nicaragua Puts out Forest Fire in Southern Nature Reserve. https://apnews.com/.

[12] Romero E. (2018). Policía Presenta a Campesino Como Autor del Incendio de la Reserva Indio Maíz. https://www.laprensa.com.ni/2018/04/18/nacionales/2405640-policia-presenta-a-campesino-como-autor-del-incendio-de-la-reserva-indio-maiz/.

[13] Waigl C., Stuefer M., Prakash A., Ichoku C. (2017). Detecting high and low-intensity fires in Alaska using VIIRS I-band data: An improved operational approach for high latitudes. Remote Sens. Environ..

[14] Potapov P., Hansen M.C., Stehman S.V., Loveland T.R., Pittman K. (2008). Combining MODIS and Landsat imagery to estimate and map boreal forest cover loss. Remote Sens. Environ..

[15] Röder A., Hill J., Duguy B., Alloza J., Vallejo R. (2008). Using long time series of Landsat data to monitor fire events and post-fire dynamics and identify driving factors. A case study in the Ayora region (eastern Spain). Remote Sens. Environ..

[16] White J., Ryan K., Key C., Running S. (1996). Remote Sensing of Forest Fire Severity and Vegetation Recovery. Int. J. Wildland Fire.

[17] Pilar M., Ceccato P., Flasse S., Downey I. (1999). Fire detection and fire growth monitoring using satellite data. Remote Sensing of Large Wildfires.

[18] European Space Agency Introducing Sentinel 3. https://www.esa.int/Our_Activities/Observing.../Sentinel-3/Introducing_Sentinel-3.

[19] European Space Agency (2015). Sentinel 3—Data Access and Products. https://sentinels.copernicus.eu/documents/247904/1848151/Sentinel-3_SLSTR_Data_Access_and_Products.pdf.

[20] European Space Agency (2018). Sentinel 3 Coverage. https://sentinel.esa.int/web/sentinel/user-guides/sentinel-3-slstr/coverage.

[21] Ulloa N.I., Chiang S.H. Mapping Forest Burned Areas in the Indio Maiz Biological Reserve Using SENTINEL-3 Slstr Imagery. Proceedings of the American Geophysical Union Fall Meeting 2018.

[22] Lee T., Miller S., Turk J., Schueler C., Julian R., Deyo S., Dills P., Wang S. (2006). The NPOESS VIIRS Day/Night Visible Sensor. Bull. Am. Meteorol. Soc..

[23] Hillger D. (2013). First-Light Imagery from Suomi NPP VIIRS. Bull. Am. Meteorol. Soc..

[24] Miller S., Straka W., Mills S., Elvidge C., Lee T., Solbrig J., Walther A., Heidinger A., Weiss S. (2013). Illuminating the Capabilities of the Suomi National Polar-Orbiting Partnership (NPP) Visible Infrared Imaging Radiometer Suite (VIIRS) Day/Night Band. Remote Sens..

[25] Osher S., Sethian J. (1988). Fronts propagating with curvature-dependent speed: Algorithms based on Hamilton-Jacobi formulations. J. Comput. Phys..

[26] Malladi R., Sethian J. Level set and fast marching methods in image processing and computer vision. Proceedings of the 3rd the IEEE International Conference on Image Processing.

[27] Plan de Manejo Reserva Biologica Indio Maiz. http://www.bio-nica.info/Biblioteca/FUNDAR2004.pdf.

[28] Munera C. (2014). Biocultural Design As a Framework to Identify Sustainability Issues in Rio San Juan Biosphere Reserve and Fortress of the Immaculate Conception, Nicaragua.

[29] Finley-Brook M. (2007). Green Neoliberal Space: The Mesoamerican Biological Corridor. J. Lat. Am. Geogr..

[30] Independent Evaluation Group (2011). The Mesoamerican Biological Corridor: Regional Program Review.

[31] Douglas G. (2011). Mesoamerican Biological Corridor: Mexico to Panama. http://www.tbpa.net/docs/62_Meso_American_Biological_Corridor.pdf.

[32] Meyer A., Huete-Perez J. (2014). Conservation: Nicaragua Canal could wreak environmental ruin. Nature.

[33] Morales S., Zolotoff J., Gutierrez M., Torrez M. (2009). Nicaragua. Important Bird Areas Americas—Priority Sites for Biodiversity Conservation.

[34] Condit R. (2015). Extracting Environmental Benefits from a New Canal in Nicaragua: Lessons from Panama. PLoS Biol..

[35] Serrano-Sandi J., Bonilla-Murillo F., Sasa M. (2013). Distribution, surface and protected area of palm-swamps in Costa Rica and Nicaragua. Rev. Biol. Trop..

[36] Roger J., Vermote E., Ray J. (2015). MODIS Surface Reflectance User’s Guide Collection 6. https://modis-land.gsfc.nasa.gov/pdf/MOD09_UserGuide_v1.4.pdf.

[37] Immitzer M., Vuolo F., Atzberger C. (2016). First Experience with Sentinel-2 Data for Crop and Tree Species Classifications in Central Europe. Remote Sens..

[38] Clark M. (2017). Comparison of simulated hyperspectral HyspIRI and multispectral Landsat 8 and Sentinel-2 imagery for multi-seasonal, regional land-cover mapping. Remote Sens. Environ..

[39] Li J., Roy D. (2017). A Global Analysis of Sentinel-2A, Sentinel-2B and Landsat-8 Data Revisit Intervals and Implications for Terrestrial Monitoring. Remote Sens..

[40] European Space Agency (2018). Sentinel 3. https://earth.esa.int/web/guest/missions/esa-eo-missions/sentinel-3.

[41] European Space Agency (2013). Sentinel-3 User Handbook. https://sentinels.copernicus.eu/documents/247904/685236/Sentinel-3_User_Handbook.

[42] Donlon C., Berruti B., Buongiorno A., Ferreira M., Fermenias P., Frerick J., Goryl P., Klein U., Laur H., Mavrocordatos C. (2012). The Global Monitoring for Environment and Security (GMES) Sentinel-3 mission. Remote Sens. Environ..

[43] Malenovský Z., Rott H., Cihlar J., Schaepman M., Garcia-Santo G., Fernandes R., Berger M. (2012). Sentinels for science: Potential of Sentinel-1, -2, and -3 missions for scientific observations of ocean, cryosphere, and land. Remote Sens. Environ..

[44] Wooster M., Xu W., Nightingale T. (2012). Sentinel-3 SLSTR active fire detection and FRP product: Pre-launch algorithm development and performance evaluation using MODIS and ASTER datasets. Remote Sens. Environ..

[45] Rouse J.W., Haas R.H., Schell J.A., Deering D.W. Monitoring Vegetation Systems in the Great Plains with ERTS. Proceedings of the Third Earth Resources Technology Satellite-1 Symposium.

[46] Chen W., Moriya K., Sakai T., Koyama L., Cao C. (2016). Mapping a burned forest area from Landsat TM data by multiple methods. Geomat. Nat. Hazards Risk.

[47] Gao B. (1996). NDWI—A normalized difference water index for remote sensing of vegetation liquid water from space. Remote Sens. Environ..

[48] Liu Y., Dai Q., Liu J., Liu S., Yang J. (2014). Study of Burn Scar Extraction Automatically Based on Level Set Method using Remote Sensing Data. PLoS ONE.

[49] Miller J., Yool S. (2002). Mapping forest post-fire canopy consumption in several overstory types using multi-temporal Landsat TM and ETM data. Remote Sens. Environ..

[50] Phua M., Tsuyiki S., Lee J., Ghani M. (2012). Simultaneous detection of burned areas of multiple fires in the tropics using multisensor remote-sensing data. Int. J. Remote Sens..

[51] Trimble (2014). eCognition Developer 9.0 Reference Book.

[52] Machala M., Zejdova L. (2014). Forest Mapping Through Object-based Image Analysis of Multispectral and LiDAR Aerial Data. Eur. J. Remote Sens..

[53] Weih R., Riggan N. (2010). Object-based classification vs. pixel based classification: Comparative importance of multi-resolution imagery. The International Archives of the Photogrammetry, Remote Sensing and Spatial Information Sciences.

[54] Giglio L., Descloitres J., Justice C., Kaufman Y. (2003). An Enhanced Contextual Fire Detection Algorithm for MODIS. Remote Sens. Environ..

[55] Schroeder W., Olivia P., Giglio L., Csiszar I. (2014). The New VIIRS 375 m active fire detection data product: Algorithm description and initial assessment. Remote Sens. Environ..

[56] Zhang T., Wooster M., Xu W. (2017). Approaches for synergistically exploiting VIIRS I- and M-Band data in regional active fire detection and FRP assessment: A demonstration with respect to agricultural residue burning in Eastern China. Remote Sens. Environ..

[57] Liao L., Weiss S., Mills S., Hauss B. (2013). Suomi NPP VIIRS day-night band on-orbit performance. J. Geophys. Res. Atmos..

[58] Gao L., Shi W., Miao Z., Lv Z. (2018). Method Based on Edge Constraint and Fast Marching for Road Centerline Extraction from Very High-Resolution Remote Sensing Images. Remote Sens..

[59] Garrido S., Malfaz M., Blanco D. (2013). Application of the fast marching method for outdoor motion planning in robotics. Robot. Auton. Syst..

[60] Chiang C.H., Chiang P.J., Fei C.C., Liu J.S. A comparative study of implementing Fast Marching Method and A* SEARCH for mobile robot path planning in grid environment: Effect of map resolution. Proceedings of the IEEE Workshop on Advanced Robotics and Its Social Impacts.

[61] Jbabdi S., Bellec P., Toro R., Daunizeau J., Pelegrini-Issac M., Benali H. (2008). Accurate anisotropic fast marching for diffusion-based geodesic tractography. J. Biomed. Imaging.

[62] Sifakis E., Tziritas G., Osher S., Paragios N. (2003). Fast Marching Techniques for Visual Grouping and Segmentation. Geometric Level Set Methods in Imaging, Vision, and Graphics.

[63] (2018). Incendio en Indio Maíz afectó 2 de las 8 Áreas Protegidas de la Reserva de Biosfera Río San Juan. El Nuevo Diario. https://www.elnuevodiario.com.ni.

[64] Verhegghen A., Eva H., Ceccherini G., Achard F., Gond V., Gourlet-Fleury S., Cerutti P. (2016). The Potential of Sentinel Satellites for Burnt Area Mapping and Monitoring in the Congo Basin Forests. Remote Sens..

[65] Urquhart G. (1999). Long-term Persistence of Raphia taedigera Mart. Swamps in Nicaragua. Biotropica.

[66] Zha Y., Gao J., Ni S., Shen N. (2005). Temporal filtering of successive MODIS data in monitoring a locust outbreak. Int. J. Remote Sens..

[67] Ji R., Xie B., Li D., Li Z., Zhang X. (2004). Use of MODIS data to monitor the oriental migratory locust plague. Agric. Ecosyst. Environ..

[68] Yin G., Yuan Z., Sun Y., Wang T., Zeng Z., Piao S. (2015). MODIS Based Estimation of Forest Aboveground Biomass in China. PLoS ONE.

